# Calbindin 2 (CALB2) Regulates 5-Fluorouracil Sensitivity in Colorectal Cancer by Modulating the Intrinsic Apoptotic Pathway

**DOI:** 10.1371/journal.pone.0020276

**Published:** 2011-05-24

**Authors:** Leanne Stevenson, Wendy L. Allen, Irina Proutski, Gail Stewart, Louise Johnston, Karen McCloskey, Peter M. Wilson, Daniel B. Longley, Patrick G. Johnston

**Affiliations:** Drug Resistance Group, Centre for Cancer Research and Cell Biology, Queen's University Belfast, Belfast, Northern Ireland; University of Illinois at Chicago, United States of America

## Abstract

The role of the calcium binding protein, Calbindin 2 (CALB2), in regulating the response of colorectal cancer (CRC) cells to 5-Fluorouracil (5-FU) was investigated. Real-time RT-PCR and Western blot analysis revealed that CALB2 mRNA and protein expression were down-regulated in p53 wild-type and p53 null isogenic HCT116 CRC cell lines following 48 h and 72 h 5-FU treatment. Moreover, 5-FU-induced apoptosis was significantly reduced in HCT116 and LS174T CRC cell lines in which CALB2 expression had been silenced. Further investigation revealed that CALB2 translocated to the mitochondria following 5-FU treatment and that 5-FU-induced loss of mitochondrial membrane potential (Δψ_m_) was abrogated in CALB2-silenced cells. Furthermore, CALB2 silencing decreased 5-FU-induced cytochrome c and smac release from the mitochondria and also decreased 5-FU-induced activation of caspases 9 and 3/7. Of note, co-silencing of XIAP overcame 5-FU resistance in CALB2-silenced cells. Collectively, these results suggest that following 5-FU treatment in CRC cell lines, CALB2 is involved in apoptosis induction through the intrinsic mitochondrial pathway. This indicates that CALB2 may be an important mediator of 5-FU-induced cell death. Moreover, down-regulation of CALB2 in response to 5-FU may represent an intrinsic mechanism of resistance to this anti-cancer drug.

## Introduction

Colorectal cancer (CRC) is the second leading cause of cancer-related deaths in Europe and the U.S.A. 5-Fluorouracil (5-FU)-based chemotherapy regimens remain the standard treatment for CRC in both the adjuvant and advanced disease settings. However, response rates to 5-FU therapy are between 10–20% in the metastatic setting [1]. The combination of 5-FU with the topoisomerase I inhibitor, irinotecan (CPT-11), or the DNA-damaging agent, oxaliplatin, has significantly improved response rates up to 50% [2]–[3]. Novel agents, such as the monoclonal antibodies cetuximab, panitumumab (epidermal growth factor receptor inhibitors), and bevacizumab (a vascular endothelial growth factor inhibitor) have also shown beneficial effects when combined with chemotherapy [4]–[6]. Despite this, the prognosis for the majority of patients with advanced CRC remains poor due to intrinsic or acquired chemoresistance. Therefore, identification of the signaling molecules involved in mediating the response of CRC to 5-FU is required to determine the underlying mechanisms of 5-FU resistance.

Calbindin-2 (CALB2, also known as calretinin) is a 29 kDa calcium (Ca^2+^) binding protein of the EF-hand family [7], which is a family of proteins containing Ca^2+^-binding motifs composed of two helices (E and F). Ca^2+^-induced conformational changes suggest that CALB2 is likely to belong to a group of Ca^2+^ sensor proteins within this family [8]. In humans, CALB2 is primarily expressed by certain cells of the nervous system, but can also be found in ovarian cells [9]. Normal colon epithelial cells do not express CALB2, but it is found in colon carcinomas [10], cell lines derived from primary colon tumours [11] and it is a diagnostic marker for mesotheliomas [12]–[13]. The role of CALB2 in modulating neuronal excitability has been consistently demonstrated [14]. However, the physiological function of CALB2 in cancer cells remains to be elucidated.

Ca^2+^ has been identified as a messenger that coordinates endoplasmic reticulum (ER)-mitochondrial interactions that regulate apoptosis [15]. Many kinds of cellular stress are known to induce Ca^2+^ release from the ER and subsequent Ca^2+^ influx into the mitochondria resulting in loss of mitochondrial membrane potential followed by release of cytochrome c and smac [16]. Induction of ER stress has also been reported to enhance chemotherapy sensitization [17]. Mitochondrial Ca^2+^ dynamics are also involved in the regulation of cellular energy metabolism and in processes such as cell motility and neurotransmitter release. Therefore the regulation of Ca^2+^ release is under tight control, and many Ca^2+^-binding proteins, such as CALB2, may function downstream of the ER Ca^2+^ release to modulate apoptosis or other cell functions.

A DNA microarray study carried out by our group using the HGU133 plus 2.0 array (Affymetrix, UK) examined the expression profiles of p53^+/+^ HCT116 CRC cells treated with 5-FU [18]. In that study, CALB2 was identified as a potential novel regulator of 5-FU response. The aim of this study was to investigate the mechanism by which CALB2 regulates 5-FU response in CRC cells.

## Materials and Methods

### Reagents

5-FU was purchased from Sigma Chemical Co. (St. Louis, MO). Stock solutions were prepared in sterile PBS and stored at 4°C prior to use. The CALB2 antibody was purchased from Chemicon International (Temecula, CA). Poly (ADP-ribose) polymerase (PARP) antibody was purchased from PharMingen (San Diego, CA, USA). Smac/DIABLO and Cytochrome c antibodies were purchased from BD biosciences (Oxford, UK). Cytochrome c oxidase sub unit IV (Cox IV) and X-linked inhibitor of apoptosis protein (XIAP) antibodies were purchased from Cell Signaling Technology, Inc (Danvers, MA, USA). Alpha-tubulin antibody was purchased from Santa Cruz Biotechnology, Inc. (Santa Cruz, CA, USA). GAPDH was purchased from AbD Serotec (Kidlington, UK). Propidium iodide was purchased from Sigma (Poole, UK) and FITC-Annexin V was purchased from BD biosciences (Oxford, UK). A pan-caspase inhibitor, Z-VAD (OMe)-FMK, was purchased from Calbiochem (Darmstadt, Germany).

### Cell culture

Parental HCT116 and isogenic p53^−/−^ and Bax^−/−^ CRC cell lines were kindly provided by Professor Bert Vogelstein (Johns Hopkins University, Baltimore, MD). The LS174T cell line was purchased from ATCC® (CL-188™). The HCT116 cell lines were maintained in McCoy's 5A medium (Invitrogen, UK) and the LS174T cell line was maintained in Dulbecco's Modified Eagle Medium (Invitrogen, UK). Media was supplemented with 10% dialysed foetal calf serum, 50 µg/ml penicillin-streptomycin, 2 mM L-glutamine and 1 mM sodium pyruvate and incubated in 5% CO_2_ at 37°C.

### Cell viability assay

Cell viability was determined by using a 3-(4, 5-dimethylthiazol-2-yl)-2, 5-diphenyl tetrazolium bromide (MTT, Sigma) assay as described previously [19].

### Real-time RT-PCR analysis

Total RNA was isolated using RNA STAT-60 reagent (Biogenesis, Poole, UK) according to the manufacturer's instructions. Reverse transcription was carried out with 8 µg of RNA using a Moloney Murine Leukaemia Virus (M-MLV) based reverse transcription kit (Invitrogen, UK) according to the manufacturer's instructions. Real-time reverse transcription-PCR (RT-PCR) amplification was carried out as described previously [18]. Primer sequences were as follows: CALB2 (forward) 5′-GCAGAGCTGGCGCAGATC- 3′, CALB2 (reverse) 5′-GCTCATCGTACGGCCGGTTCG- 3′; GAPDH (forward) 5′ -ACAGTCAGCCGCATCTTCTT- 3′ and GAPDH (reverse) 5′ - GACAAGCTTCCCGTTCTCAG -3′. Gene expression was normalised to expression of the GAPDH reference gene. Final expression values were presented relative to the untreated time-matched control.

### Western blotting

Western blots were performed as previously described [19]. Immunodetection was performed using primary mouse monoclonal antibodies against CALB2, PARP, Smac, cytochrome c, XIAP, α-tubulin or GAPDH in conjunction with horseradish peroxidise-conjugated sheep anti-mouse antibody (Amersham, Little Chalfont, Buckinghamshire, England). A rabbit polyclonal antibody against Cox IV was used in conjunction with a donkey anti-rabbit secondary antibody (Amersham). The fluorescent signal was detected using the Super Signal chemiluminescent detection system (Pierce, Rockford, IL), according to the manufacturer's instructions.

### Western blot protein quantification

Densitometry values of protein bands were acquired using the ChemiDoc-XRS systems ship documentation system (Bio-Rad Laboratories, Inc.). Bands were then analyzed using the Quantity One®1-D analysis software (Bio-Rad Laboratories, Inc., version, 4.5.2).

### Analysis of subG1/G0

DNA content of cells was evaluated by propidium iodide (PI) staining as described previously [18]. Measurements and analysis were performed on a FACS Calibur flow cytometer with CELLQUEST software (BD Biosciences, San Diego).

### FITC Annexin V/Propidium Iodide analysis

Cells were harvested and analysed according to the manufacturer's instructions (BD Biosciences, Oxford, UK). Briefly, levels of apoptosis were calculated as the sum of FITC-Annexin V positive/propidium iodide negative (early apoptosis) and FITC-Annexin V positive/propidium iodide positive (late apoptosis) cell population. Measurements and analysis were performed on a FACS Calibur flow cytometer with CELLQUEST software (BD Biosciences, San Diego).

### Mitochondrial membrane potential analysis

To determine the loss of the mitochondrial membrane potential (Δ*ψ*_m_), cells were incubated with 25 nM tetramethylrhodamine, ethyl ester percholate (TMRE) at 37°C for 15 min and collected by trypsinisation. Cells were pelleted by centrifugation at 800 *g* for 5 min at 4°C and resuspended in 0.5 ml PBS. Measurement and analysis were performed on a FACS Calibur flow cytometer with CELLQUEST software (BD Biosciences, San Diego).

### siRNA transfection

The CALB2 targeting (siCALB2) and non-targeting control (SC) siRNA constructs were purchased from Dharmacon Inc. (Chicago, IL). The siCALB2 construct sequences used were GGCUCUGGCAUGAUGUCAAdTdT (sense) and UUGACAUCAUGCCAGAGCCdTdT (antisense). The SC construct sequences used were UUCUCCGAACGUGUCACGUdTdT (sense) and ACGUGACACGUUCGGAGAAdTdT (antisense). An additional 4 CALB2-targeting siRNA sequence were purchased from Qiagen: siCALB2_5 (SI02660980), siCALB2_6 (SI03190824), siCALB2_7 (SI04157790) and siCALB2_8 (SI04267697). Pre-designed siRNA for XIAP was purchased from Cell Signalling Technology (Danvers, MA, USA). siRNA transfections were performed with Oligofectamine reagent (Invitrogen) according to the manufacturer's instructions. After 5 h, the cells were treated with parental ∼IC_60(72 h)_ doses of 5-FU (5 µM for HCT116 and 20 µM for LS174T). Cells were harvested following 72 h 5-FU treatment prior to analysis by flow cytometry and Western blot.

### Sub-cellular fractionation

Cells were collected by centrifugation, washed in 1 ml of ice-cold mitochondrial isolation buffer (200 mM Mannitol, 70 mM sucrose, 1 mM ethylene glycol bis (α-aminoethylether)-*N*,*N*,*N^1^*,*N^1^*,-tetraacetic acid, 10 mM HEPES, 0.5 mg/ml bovine serum albumin; pH 7.4) prior to dounce homogenisation. Cell debris was collected by centrifugation at 800 *g* for 10 min. the mitochondrial fraction was pelleted by centrifugation at 10,000 *g* for 10 min. The supernatant containing the cytosolic fraction was transferred to a fresh tube and the mitochondrial pellet was resuspended in 50 µl RIPA buffer (50 mM Tris pH 7.4, 150 mM NaCl, 5 mM EDTA, 1% Triton-X100, 0.1% SDS) containing protease inhibitor cocktail (Roche Diagnostics, Mannheim, Germany).

### Caspase Activation Assays

Caspase activation was measured using Caspase Glo 3/7, 8 and 9 assays (Promega) according to the manufacturer's instructions.

### Statistical analysis

Results were summarised as a mean ± standard error of the mean (SEM) of 3 independent experiments. The statistical significance of the data was tested using a 2-way ANOVA (GraphPad PRISM® version 5.01).

### Microarray analysis

The 5-FU-resistant HCT116 sub-line was generated in our laboratory as previously described [20]. HCT116 parental cells and HCT116 5-FU resistant daughter cells were treated with 5 µM 5-FU for 24 h to identify genes that were differentially expressed. Total RNA was isolated from the *in vitro* experiments using the RNA STAT-60 Total RNA isolation reagent (Tel-Test) according to the manufacturer's instructions. Total RNA (5 µg) was sent to Almac Diagnostics for cDNA synthesis, cRNA synthesis, fragmentation, and hybridization onto the colorectal Disease Specific Array (DSA, Almac Diagnostics) microarrays. All *in vitro* assays were carried out in triplicate. All microarray data is MIAME compliant and all raw data has been deposited in a MIAME compliant database (ArrayExpress accession number: E-MEXP-1691).

### Generation of gene lists

Initially each array was normalized to the median signal intensity of all arrays. For the drug treated arrays, the 24 h 5-FU treated samples were then normalized to the untreated control samples. In the case of the basal experiment, the 5-FU-resistant arrays were normalized to the HCT116 parental arrays. All microarray data (E-MEXP-1691) was then filtered using the following criteria: Affymetrix flag calls (P/M in all samples), Cross Gene Error Model (average base/proportional cut-off), fold change (1.5-fold) and t test (p<0.05), only genes passing all 4 filters were retained and used as the final working genelists. Three *in vitro* genelists were created: 5-FU-inducible in parental, 5-FU-inducible in 5-FU-resistant and constitutively deregulated in 5-FU-resistant.

### Identification of pathways associated with 5-FU-resistance

KEGG pathway function within Genespring GX (v7.3.1) was used to identify deregulated pathways passing a 1.5 fold change and t-test (p<0.05) from the microarray *in vitro* genelists.

### Association of CALB2 expression with clinical response

We downloaded CALB2 expression data from public microarray data sets. GraphPad Prism 5 software was used to generate Kaplan-Meier survival curves based on median CALB2 expression across each tumour stage or combined staging. These data sets included a CRC cohort (GSE12945; PMID: 19399471) of 62 patients (13 stage 1, 23 stage 2, 21 stage 3 and 5 stage IV) undergoing elective standard oncological resection [21] and a breast cancer cohort (GSE9893; PMID: 18347175) of 132 tamoxifen-treated patients) [22].

## Results

### CALB2 expression in HCT116 cells is down-regulated by 5-FU treatment

**Figure 1:**
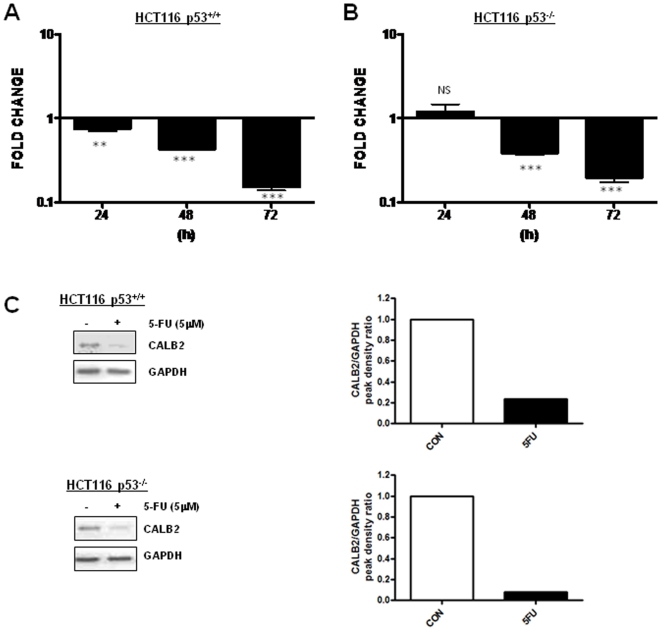
5-FU down-regulates CALB2 expression in HCT116 cell lines. Real-time RT-PCR quantification of CALB2 mRNA levels in isogenic (A) p53^+/+^ and (B) p53^−/−^ HCT116 cells following 24 h, 48 h and 72 h 5-FU treatment (parental ∼IC60_(72 h)_ dose of 5 µM). Fold change in expression values are relative to the untreated time-matched control. Error bars represent mean ± SEM, NS: no significant difference, **p<0.01, ***p<0.001. (C) Western blot analysis of CALB2 in HCT116 cell lines following 72 h 5-FU (5 µM) treatment and corresponding densitometry data of CALB2 expression relative to the GAPDH loading control.

A previous microarray study by our group reported CALB2 gene expression in the HCT116 cell line to be up-regulated following 24 h 5-FU treatment relative to an untreated 0 h control [18]. However, further analyses revealed that constitutive CALB2 expression increases over time (Fig. S1), therefore we re-analysed 5-FU-induced mRNA changes in CALB2 gene expression levels relative to an untreated, time-matched control. In contrast to our previous study, this method indicated that treatment with an ∼IC_60(72 h)_ dose (5 µM) of 5-FU actually resulted in a significant, time-dependent down-regulation of CALB2 gene expression in the p53^+/+^ HCT116 cell line (Fig. 1A). In the p53^−/−^ HCT116 cell line, CALB2 gene expression was not significantly altered after 24 h 5-FU treatment, but was significantly down-regulated at 48 h and 72 h (Fig. 1B). Western blot analysis demonstrated that this down-regulation was reflected in reduced CALB2 protein expression in 5-FU treated cells (Fig. 1C). These results suggest that CALB2 expression in HCT116 cells is acutely down-regulated in response to 5-FU treatment and that this modulation is not dependent on p53.

### 5-FU induced down-regulation of CALB2 is caspase-independent

**Figure 2:**
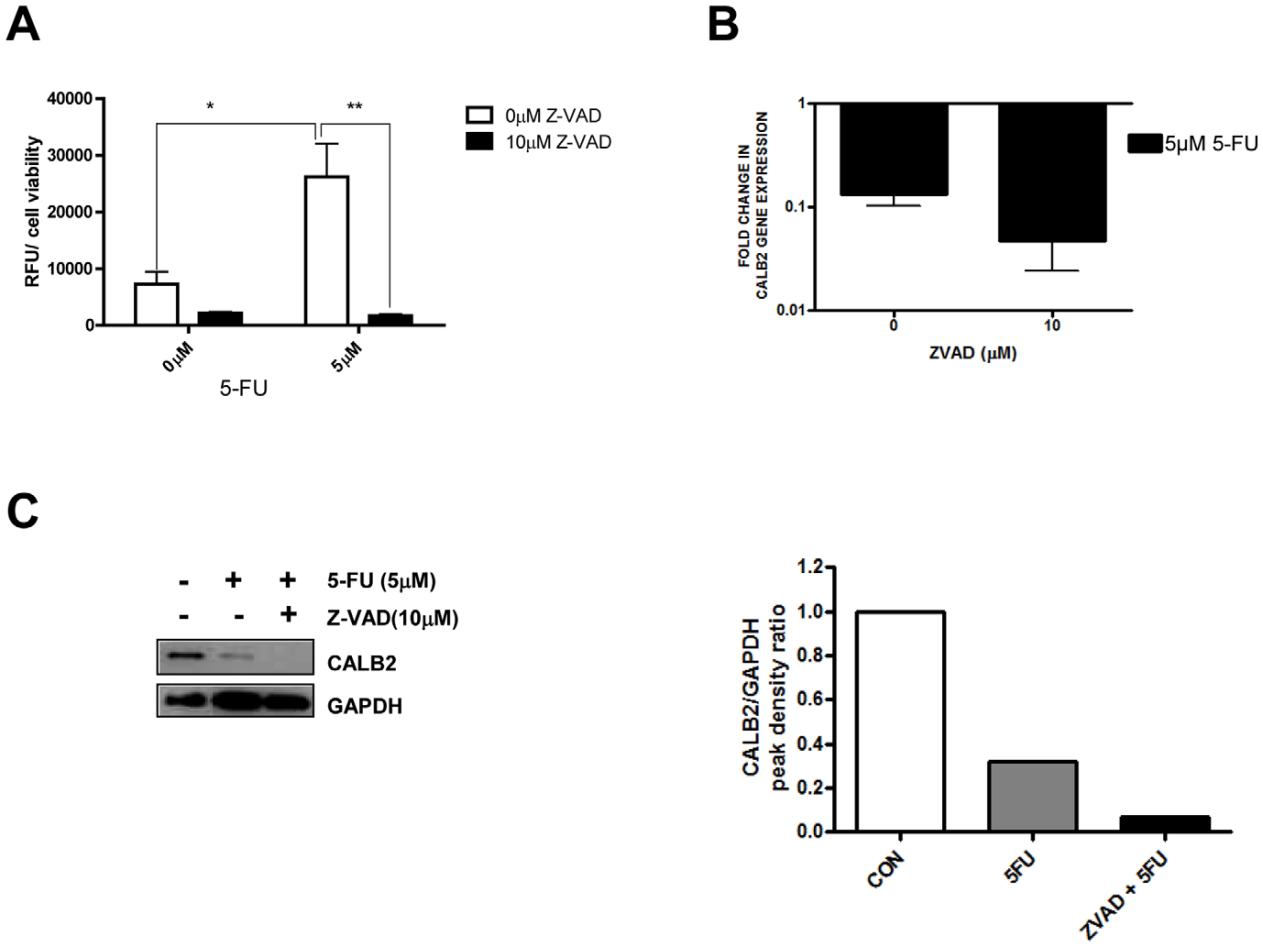
5-FU-induced down-regulation of CALB2 is caspase-independent. p53^+/+^ HCT116 cells were treated with an ∼IC60_(72 h)_ dose of 5-FU alone or combined with a 10 µM dose of pan-caspase inhibitor Z-VAD (OMe)-FMK (Z-VAD) for 72 h (A) Caspase 3/7 activity was quantified using a luciferase reporter based assay. Values are RFU read-outs relative to cell viability, as determined by MTT. (B) Real-time RT-PCR quantification of CALB2 mRNA (fold-change relative to the untreated, time-matched control). (C) Western blot analysis of CALB2 expression and corresponding densitometry data of CALB2 expression relative to the GAPDH loading control.

To determine whether the decrease in CALB2 protein in response to 5-FU was caspase-dependent, HCT116 parental cells were treated with an ∼IC_60(72 h)_ dose (5 µM) of 5-FU alone or in combination with a 10 µM dose of a pan-caspase inhibitor (Z-VAD) for 72 h. Inhibition of caspase activity following Z-VAD treatment was validated using a caspase 3/7-specific activity assay (Fig. 2A). 5-FU treatment significantly (p<0.05) increased caspase 3/7 activity by ∼4 fold, compared to the untreated controls, and this was completely abrogated by Z-VAD. Importantly, Z-VAD co-treatment did not inhibit 5-FU-induced down-regulation of CALB2 gene expression (Fig. 2B) or protein expression (Fig. 2C), indicating that CALB2 down-regulation is not caspase-dependent.

### CALB2 silencing confers 5-FU resistance

**Figure 3:**
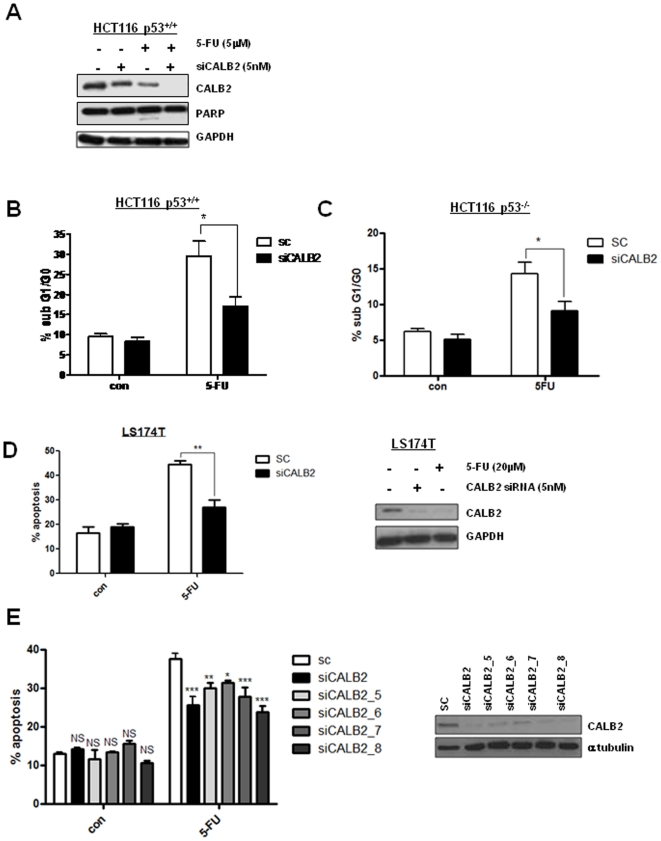
CALB2 silencing confers 5-FU resistance in CRC cell lines. (A) Western blot analysis of CALB2 and PARP in the p53^+/+^ HCT116 cell line following transfection with control siRNA (−) or CALB2-targeting siRNA (+) and 72 h co-treatment with ∼IC60_(72 h)_ dose of 5-FU. GAPDH was used as a loading control. Propidium Iodide flow cytometric analysis showing the percentage of cell population in sub-G1/G0 for (B) p53^+/+^ and (C) p53^−/−^ HCT116 cells following transfection with control siRNA (SC) or CALB2 targeting siRNA (siCALB2) and 72 h co-treatment with 5 µM 5-FU. (D) Annexin V/propidium iodide flow cytometric analysis of the LS174T CRC cell line following transfection with control siRNA (SC) or CALB2 targeting siRNA (siCALB2) and 72 h co-treatment with ∼IC60_(72 h)_ dose of 5-FU (20 µM). ** p<0.01, error bars represent mean ± SEM. (Inset) Western blot analysis of CALB2 expression in LS174T. GAPDH was used as a loading control. (E) p53^+/+^ HCT116 cells following transfection with control siRNA (SC), CALB2 targeting siRNA (siCALB2) or additional CALB2-targeting siRNA sequences (siCALB2_5, siCALB2_6, siCALB2_7 and siCALB2_8) and 72 h co-treatment with 5 µM 5-FU. (Inset) Western blot analysis of CALB2 expression following 72 h transfection with control siRNA (SC) or CALB2 targeting siRNAs in p53^+/+^ HCT116 cells. α-tubulin was used as a loading control.

Since CALB2 is down-regulated following 5-FU treatment, we investigated its role in mediating 5-FU-response. A gene silencing approach was used to determine the function of CALB2 in 5-FU-induced apoptosis. CALB2 knock down by siRNA was confirmed by Western blot (Fig. 3A). siRNA-mediated silencing of CALB2 expression was further enhanced by 5-FU co-treatment, most likely due to the suppression of CALB2 mRNA in response to 5-FU treatment (Fig. 1A). PARP cleavage, an indicator of cell death, was observed in the control siRNA-transfected p53^+/+^ HCT116 cells following 5-FU treatment. This was completely abrogated in the CALB2-silenced cells, indicating reduced 5-FU-induced death. These results were supported by flow cytometry data which indicated that the level of 5-FU-induced apoptosis was significantly reduced (p<0.05) from ∼30% in the siRNA control cells to ∼17% in the CALB2-silenced p53^+/+^ HCT116 cells (Fig. 3B). In the p53^−/−^ HCT116 cell line, 5-FU-induced apoptosis was also significantly reduced (p<0.05), from ∼14% in the siRNA control cells to ∼9% in the CALB2-silenced cells (Fig. 3C). The reduced sensitivity of p53^−/−^ HCT116 cells to 5-FU has been previously noted by our group and others [23], [24]. Similar effects were observed in another CRC cell line, LS174T, where apoptosis was significantly increased following an ∼IC_60(72 h)_ dose of 20 µM 5-FU and CALB2 silencing significantly reduced this (Fig. 3D, p<0.01). CALB2 knock down was confirmed by Western blot (Fig. 3D inset) and as in the HCT116 cell lines, CALB2 protein levels were down-regulated following 5-FU treatment. To rule out off-target effects of the CALB2-targeting siRNA, we used Annexin/PI flow cytometry to determine the effect of an additional 4 CALB2-targeting siRNA sequences on 5-FU-induced apoptosis in the p53^+/+^ HCT116 cells (Fig. 3E). A significant reduction in 5-FU-induced apoptosis was observed with all 4 additional CALB2 siRNA sequences and CALB2 silencing was confirmed by Western blot analysis (inset).

### 5-FU resistance is associated with de-regulation of Ca^2+^ signalling

The identification of CALB2's role in mediating 5-FU-induced apoptosis together with its Ca^2+^ binding property led us to investigate the effect of 5-FU on Ca^2+^ signalling in general. Transcriptional profiling experiments were carried out in HCT116 parental and 5-FU-resistant daughter cells lines pre- and post-treatment with 5 µM 5-FU for 24 h. Microarray analysis identified 1389 genes in the HCT116 parental cells and 922 genes in the 5-FU-resistant daughter cells that were altered (≥1.5-fold, p<0.05) following 5-FU treatment. Also, 1329 genes were identified as constitutively altered (≥1.5-fold, p<0.05) between the HCT116 parental cells and the 5-FU-resistant daughter cells. The resultant genelists were then used for pathway analysis using the KEGG pathway function within Genespring GX (v7.3.1). Pathway analysis identified 60 pathways in the HCT116 parental cells and 24 pathways in the 5-FU-resistant daughter cells that were altered (≥1.5-fold, p<0.05) following 24 h 5 µM 5-FU treatment. Also, 49 pathways were identified as constitutively altered (≥1.5-fold, p<0.05) between the HCT116 parental cells and the 5-FU-resistant daughter cells (Table S1). The results from the pathway analysis demonstrated that 11 pathways were commonly altered in each of the three genelists (Table 1). Of particular relevance to the current study were the Ca^2+^ signalling and apoptosis pathways, which were altered in both the parental and 5-FU-resistant cells following 5-FU treatment and also basally deregulated in 5-FU-resistant cells when compared to the parentals. This suggests that Ca^2+^ signalling and apoptosis may be mediators of 5-FU sensitivity/resistance in this setting.

**Table 1:** Pathways associated with 5-FU resistance.

	Number of genes passing filter of 1.5 fold-change and p<0.05		
	Inducible parental	Inducible resistant	Constitutively de-regulated
Apoptosis	7	7	5
Calcium signalling	8	7	9
Cell cycle	21	13	11
Focal adhesion	16	13	10
Insulin signalling	20	12	8
MAPK signalling	19	14	12
Purine metabolism	19	12	17
Pyrimidine metabolism	9	12	10
Regulation of actin cytoskeleton	15	16	8
Tight junction	6	14	7
Wnt signalling	12	8	8

KEGG pathway analysis was used to identify pathways from 5-FU *in vitro* genelists and the pathways common to all genelists were selected. The 5-FU *in vitro* genelists were: (1) genes induced by 5-FU in the sensitive HCT116 parentals (inducible parental), (2) genes induced by 5-FU in the 5-FU-resistant HCT116 sub-line (inducible resistant) and (3) constitutively de-regulated genes in the 5-FU-resistant sub-line compared to the sensitive parentals (constitutively deregulated). Pathways contained genes passing a 1.5 fold change and t-test (p<0.05).

### CALB2 silencing abrogates 5-FU-induced apoptosis through the intrinsic pathway

**Figure 4:**
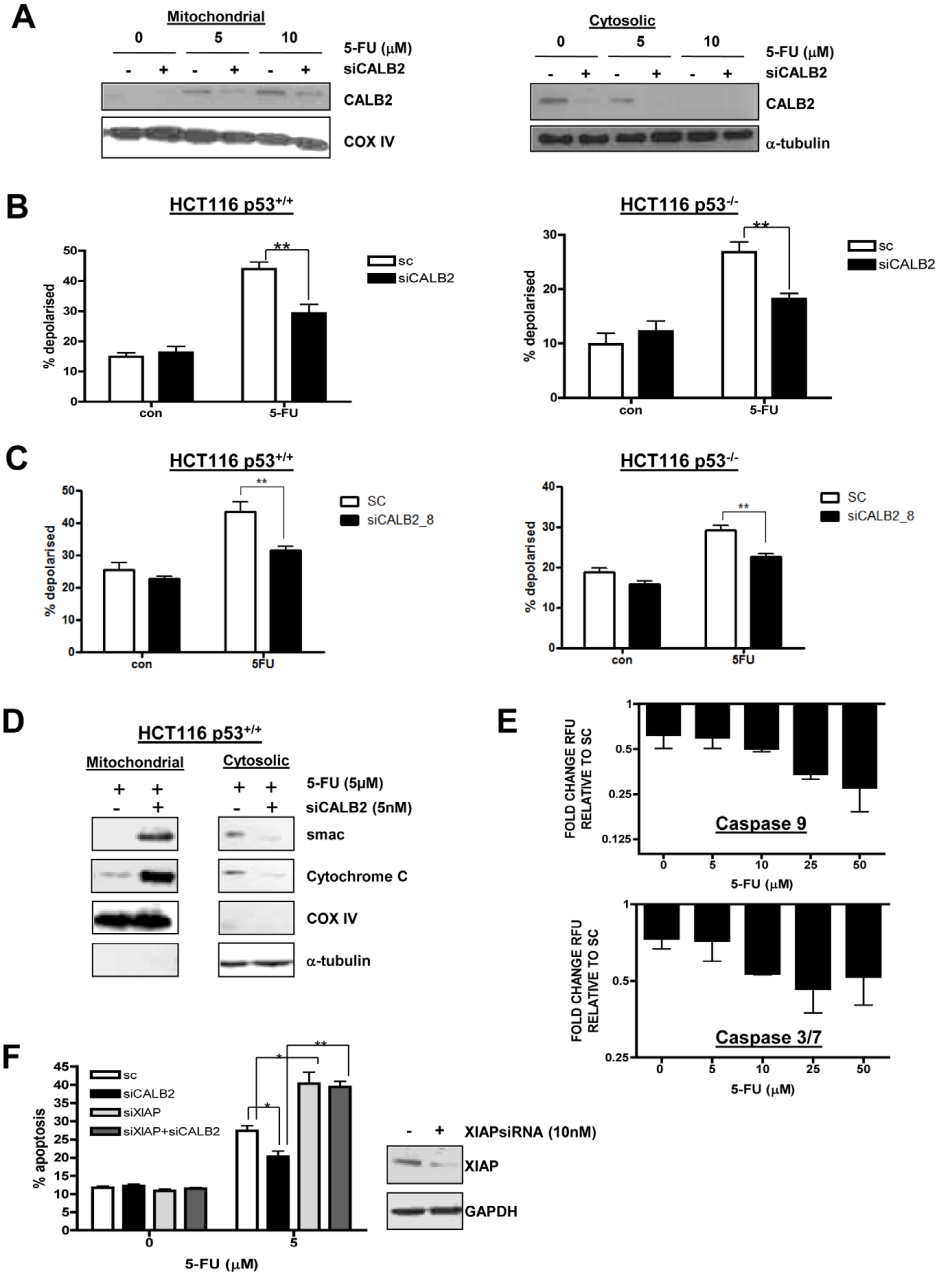
Increased 5-FU resistance in CALB2 silenced cells is associated with abrogated induction of the intrinsic apoptotic pathway. (A) Western blot analysis of CALB2 in the mitochondrial and cytosolic fractions of p53^+/+^ HCT116 cells following transfection with control siRNA (−) or CALB2-targeting siRNA (+) and 72 h co-treatment with 5-FU. Cox IV was used as a mitochondrial loading control and α-tubulin was used as a cytosolic loading control. (B) TMRE flow cytometric analysis showing the percentage of isogenic p53^+/+^ and p53^−/−^ HCT116 cells with loss of Δψ_m_ (% depolarised) following transfection with control siRNA (sc) or CALB2 targeting siRNA (siCALB2) and 72 h co-treatment with 5 µM 5-FU. **p<0.01, error bars represent mean ± SEM. (C) TMRE flow cytometry experiment repeated with a second siCALB2 sequence. (D) Western blot analysis of smac and cytochrome C in the mitochondrial and cytosolic fractions of 5-FU treated p53^+/+^ HCT116 cells. Cox IV and α-tubulin were used as loading controls. (E) Caspase activity assays in p53^+/+^ HCT116 cells. RFU values were normalised to cell viability (MTT assay) and expressed as fold-change RFU in the CALB2 silenced cells relative to the siRNA control (SC) cells. Error bars represent the mean ± SEM. (F) Annexin V/propidium iodide flow cytometric analysis of apoptotic p53^+/+^ HCT116 cells following CALB2 or XIAP silencing alone or in combination. Cells were co-treated with 5 µM 5-FU for 72 h as indicated. *p<0.05, **p<0.01, error bars represent mean ± SEM. (Inset) Western blot analysis of XIAP expression following transfection with 10 nM control siRNA (−) or 10 nM XIAP targeted siRNA (+). GAPDH was used as a loading control.

Given the Ca^2+^ binding properties of CALB2 and the integral role of Ca^2+^ signalling in the intrinsic apoptotic pathway, the role of CALB2 in mediating 5-FU-induced, mitochondrial-regulated apoptosis was investigated further. Examination of the HCT116 sub-cellular fractions demonstrated that in untreated cells, CALB2 was located in the cytosol and was not associated with the mitochondria (Fig. 4A). However, following 72 h 5-FU treatment, CALB2 levels in the cytosol decreased whilst mitochondrial CALB2 levels increased, indicating 5-FU-induced CALB2 translocation to the mitochondria. Western blot analysis of α-tubulin and CoxIV demonstrated the purity of these sub-cellular fractions (Fig. S2). These results demonstrate an inducible association between CALB2 and the mitochondria in response to 5-FU treatment.

The association between CALB2 and the mitochondria following 5-FU treatment prompted us to further investigate the role of the intrinsic apoptotic pathway in 5-FU resistance. The pro-apoptotic Bcl-2 family member, Bax is an important initiator of mitochondrial-mediated apoptosis. We therefore examined HCT116 parental and isogenic Bax null cells following treatment with the parental ∼IC_60(72 h)_ dose of 5-FU for 72 h. The parental ∼IC_60(72 h)_ dose of 5-FU caused an ∼3-fold increase in apoptosis in the HCT116 parental cell line (p<0.01), but had no significant effect on levels of apoptosis in the Bax null cells (data not shown). These results confirm previous findings [25], [26] that Bax and the intrinsic apoptotic pathway are major effectors of 5-FU-induced apoptosis.

Loss of mitochondrial membrane potential (Δ*ψ*_m_) with concomitant release of smac and cytochrome c followed by caspase 9 and 3/7 activation is observed during apoptosis via the intrinsic pathway. Therefore, TMRE, a fluorescent dye for measuring membrane potential of mitochondria was used to determine Δ*ψ*_m_ by flow cytometry (Fig. 4B). 5-FU-induced loss of Δ*ψ*_m_, as indicated by non-fluorescent cells, was significantly reduced (p<0.001) in the CALB2-silenced p53^+/+^ HCT116 cells. 5-FU-induced loss of Δ*ψ*_m_ was also significantly reduced (p<0.05) in the CALB2-silenced p53^−/−^ cells. This effect was confirmed with a second CALB2 siRNA sequence (Fig. 4C), ruling out off-target effects of the CALB2 siRNA. In addition, Western blot analysis of sub-cellular fractions revealed that 5-FU-induced loss of smac and cytochrome c from the mitochondrial fraction was abrogated in CALB2-silenced cells (Fig. 4D). This was concurrent with reduced levels of smac and cytochrome c released into the cytosol in response to 5-FU in the CALB2-silenced cells. Furthermore, 5-FU-induced activation of caspase 9 and 3/7, as measured by luciferase reporter-based activity assays, was significantly reduced in the CALB2-silenced cells (Fig. 4E). No significant differences in caspase 8 activation were observed between the CALB2-silenced cells and siRNA controls following 72 h 5 µM 5-FU treatment (data not shown). These results further suggest a role for CALB2 in regulating the 5-FU-induced, intrinsic apoptotic pathway.

### 5-FU sensitivity in CALB2-silenced cells is restored by XIAP co-silencing

The experiments described above demonstrate that maintenance of mitochondrial membrane potential, retention of smac and cytochrome c by the mitochondria with concomitant reduction in caspase activity are all associated with increased 5-FU resistance in CALB2-silenced cells, suggesting dysfunctional signalling via the intrinsic apoptotic pathway. A previous study by our group demonstrated that apoptosis resistance conferred by a compromised intrinsic apoptotic pathway can be bypassed following XIAP silencing. XIAP inhibits caspase 9 activation through its BIR3 domain and caspase 3 activation through its BIR2 domain. Smac release disrupts the interaction of XIAP with caspase 9 and caspase 3 [27], leading to activation of these caspases and subsequent apoptosis. We hypothesised that silencing XIAP may overcome resistance to 5-FU caused by CALB2 silencing by bypassing the block in the intrinsic apoptotic pathway. Therefore, the effect of co-silencing XIAP and CALB2 on 5-FU response was investigated. XIAP silencing in HCT116 cells using a XIAP-targeting siRNA was confirmed by Western blot (Fig. 4F inset). The apoptotic fraction of cells was identified by flow cytometry with Annexin V and propidium iodide. This again demonstrated that 5-FU-induced apoptosis was significantly reduced (p<0.05) in CALB2-silenced cells (Fig. 4F) whereas, 5-FU induced apoptosis was significantly increased in XIAP-silenced cells (p<0.05). Importantly, the resistance to 5-FU-induced apoptosis conferred by CALB2 silencing was overcome by co-silencing XIAP (p<0.01). Of note, CALB2 silencing did not affect XIAP levels (Fig. S3). These results suggest that XIAP silencing abrogates 5-FU resistance caused by CALB2 silencing.

### CALB2 expression in CRC patients

**Figure 5:**
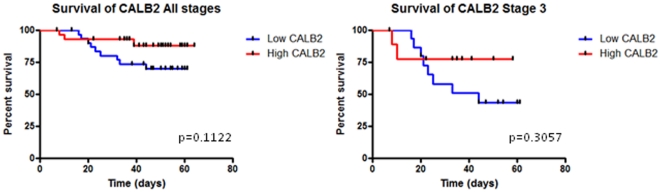
CALB2 expression in CRC patients. Kaplan-Meier survival curves for CALB2 expression across a CRC patient cohort (Dataset: GSE12945, probe ID: 20542 s at, n = 62). CALB2 expression and patient survival were analysed for stage 3 patients (n = 26, includes 5 stage 4 patients) and combined stage tumours (n = 62). The red line shows patients with high CALB2 expression and the blue line shows patients with low CALB2 expression. High and low level expression cut-points were based on median CALB2 expression.

Given the pro-apoptotic function of CALB2 in CRC cell lines, we used a public CRC data set to determine whether CALB2 expression was associated with patient prognosis (Fig. 5). CALB2 expression in the CRC GSE12945 dataset (PMID: 19399471; probe ID: 20542 s at) was analysed for stage 3 tumours and combined staging. A non-significant trend for high CALB2 expression correlating with increased overall survival was observed in all patients (HR = 2.507; 95% CI of ratio = 0.8066–7.793; p = 0.1122; n = 62) and the later stage patients (p value = 0.3057; n = 26, includes 21 stage 3 and 5 stage 4 patients). This data suggests a potential association between CALB2 and patient outcome, however this would require validation in a large independent CRC patient cohort.

The association between CALB2 expression and patient survival was also examined in the GSE9893 breast cancer dataset (PMID: 18347175). In this data set (132 patients), high CALB2 expression (probe ID: 20864) was significantly associated with increased overall survival (hazard ratio 1.86; 95% CI of ratio = 1.054–3.285; minimum p value = 0.001826; corrected p value = 0.0322, Fig. S3), suggesting that CALB2 expression may have a prognostic role in this disease.

## Discussion

We previously identified CALB2 as a potential regulator of 5-FU resistance following a DNA microarray profiling experiment [18]. The aim of this study was to further investigate the role of CALB2 in regulating 5-FU response in CRC cells. CALB2 transcription and protein expression were down-regulated in p53 wild-type (WT) and null HCT116 cell lines following 5-FU treatment Down-regulation of CALB2 expression was not abrogated by the pan-caspase inhibitor Z-VAD, demonstrating that this process is independent of caspase activity.

In order to determine the role of CALB2 in the regulation of 5-FU response, gene silencing studies were conducted. CALB2 silencing significantly increased 5-FU resistance in the HCT116 cell lines, both p53 WT and null, and the LS174T cell line which is p53 WT, but does not express p53 (www.lgcstandards-atcc.org). These results indicate a pro-apoptotic role for CALB2 in 5-FU-mediated cell death and demonstrate that this process may be independent of p53 status. *In vitro* studies based on neuroprotection, report contradictory roles for CALB2, with some studies suggesting an anti-apoptotic role and others reporting no effect. In the P19 murine model, cells that were stably transfected with CALB2 cDNA exhibited delayed onset of cell death induced by excitotoxic stimulation [28]. A follow-on study using an N18-RE 105 neuroblastoma-retina hybrid model, demonstrated that cells stably transfected with CALB2 cDNA were protected from Ca^2+^-dependent L-glutamate-induced cytotoxicity [29]. In contrast to the protective role of CALB2, a lack of protection from Ca^2+^ overload and trophic factor deprivation was reported in rat PC12 cells with either exogenous or endogenous CALB2 expression [30]. The present study proposes a pro-apoptotic role for CALB2 which may highlight cancer-specific functions for CALB2 that are relevant in the context of chemotherapy treatment. In addition, acute down-regulation of CALB2 in response to 5-FU treatment may represent a mechanism of 5-FU resistance in cancer cells.

Having identified CALB2 as a modifier of 5-FU sensitivity, the mechanism by which CALB2 silencing confers 5-FU resistance was investigated. Given the Ca^2+^ binding property of CALB2, we hypothesised that Ca^2+^ signalling is important in mediating response of CRC cells to 5-FU. Pathway analysis of microarray data from the HCT116 *in vitro* model indicated that the Ca^2+^ signalling and apoptosis pathways were altered in the 5-FU resistant HCT116 cells compared to the 5-FU sensitive parentals. Since Ca^2+^-mediated crosstalk between the ER and the mitochondria determines the balance between cell survival and mitochondrial-mediated cell death [16], these findings prompted us to investigate the role of CALB2 in the intrinsic apoptotic pathway.

CALB2 was found to translocate to the mitochondria following 5-FU treatment suggesting a role in regulating Ca^2+^ signalling to the mitochondria. The present study also demonstrated that 5-FU sensitivity was compromised in the Bax^−/−^ cells compared to the parental HCT116 cells (data not shown). This further confirms a role for the intrinsic apoptotic pathway in 5-FU resistance and is in agreement with previous studies [25], [26].

Loss of mitochondrial membrane potential (Δ*ψ*_m_) followed by release of mitochondrial pro-apoptotic proteins such as smac and cytochrome c into the cytosol is a critical step during mitochondrial-regulated apoptosis [31] and leads to caspase activation. We demonstrated that CALB2 silencing inhibits this cascade of events induced by 5-FU treatment and implicate CALB2 as a positive mediator of the intrinsic apoptotic pathway. 5-FU sensitivity was restored upon XIAP co-silencing in CALB2-silenced cells. This is in agreement with a previous study by our group [26] which used the Bax^−/−^ cells to show that XIAP silencing could re-sensitise cells with a dysfunctional intrinsic apoptotic pathway to FLIP silencing. This result further suggests dysfunctional intrinsic apoptotic signalling as a mechanism for 5-FU resistance in CALB2-silenced cells.

Given the pro-apoptotic function of CALB2, we hypothesised that cancer patients with higher levels of CALB2 would have a better prognosis. Investigation of CALB2 expression levels in patients within a CRC cohort [21] showed that patients with high levels of CALB2 had a better overall survival, although this was not significant (p = 0.1122). When sub-divided according to tumour stage, this trend was also observed in stage 3 patients. Despite the lack of chemotherapy information available for this cohort, it is likely that the stage 3 patients will have received 5-FU based chemotherapy and this may therefore suggest an association of CALB2 expression with 5-FU response. High CALB2 levels were also associated with better overall survival in a breast cancer cohort treated with tamoxifen and radiotherapy [22] (Fig. S4, p = 0.0322). Overall, these results are consistent with our findings that CALB2 has a pro-apoptotic role following chemotherapy and suggests that CALB2 may be a potentially useful prognostic biomarker. However, this would require substantial verification.

In conclusion, this study identifies a novel role for CALB2 as a modulator of 5-FU-induced death in CRC cells through activation of the intrinsic apoptotic pathway.

## Supporting Information

Figure S1 **CALB2 gene expression in untreated HCT116 cells.** Real-time RT-PCR quantification of CALB2 mRNA levels in untreated p53^+/+^ HCT116 cells following 24 h, 48 h and 72 h culture. Fold change in expression values are relative to the untreated 0 h control. Error bars represent mean ± SEM, **p<0.01.
(TIF)

Figure S2 **Western blot analysis of CoxIV and α-tubulin levels in p53^+/+^ HCT116 mitochondrial and cytosolic fractions.**
(TIF)

Figure S3 **Western blot analysis of XIAP expression following 72 h transfection with 5 nM control siRNA (−) or CALB2 targeted siRNA (+). GAPDH was used as a loading control.**
(TIF)

Figure S4 **CALB2 expression in breast cancer.** Kaplan-Meier survival curve across a breast cancer patient cohort (Dataset: GSE9893, probe ID: 20864; n = 132; HR = 1.861; CI of ratio = 1.054–3.285; p = 0.0322; n = 132). The red line shows patients with high CALB2 expression and the blue line shows patients with low CALB2 expression. High and low level expression cut-points were based on median CALB2 expression.
(TIF)

Table S1 **Identification of de-regulated pathways associated with 5-FU resistance by pathway analysis.** KEGG pathway analysis was used to identify pathways associated with 5-FU resistance from the 5-FU *in vitro* genelists: (1) genes induced by 5-FU in the sensitive HCT116 parentals (inducible parental), (2) genes induced by 5-FU in the 5-FU-resistant HCT116 sub-line (inducible resistant) and (3) constitutively de-regulated genes in the 5-FU-resistant sub-line compared to the sensitive parentals (constitutively deregulated). All pathways contained ≥5 genes passing a 1.5 fold change and t-test (p<0.05).
(DOCX)

## References

[1] Johnston PG, Kaye S (2001). Capecitabine: A novel agent for the treatment of solid tumors.. Anticancer Drugs.

[2] Douillard JY, Cunningham D, Roth AD, Navarro M, James RD (2000). Irinotecan combined with fluorouracil compared with fluorouracil alone as first-line treatment for metastatic colorectal cancer: A multicentre randomised trial.. Lancet.

[3] Giacchetti S, Perpoint B, Zidani R, Le Bail N, Faggiuolo R (2000). Phase III multicenter randomized trial of oxaliplatin added to chronomodulated fluorouracil-leucovorin as first-line treatment of metastatic colorectal cancer.. J Clin Oncol.

[4] Cunningham D, Humblet Y, Siena S, Khayat D, Bleiberg H (2004). Cetuximab monotherapy and cetuximab plus irinotecan in irinotecan-refractory metastatic colorectal cancer.. N Engl J Med.

[5] Caponigro F, Formato R, Caraglia M, Normanno N, Iaffaioli RV (2005). Monoclonal antibodies targeting epidermal growth factor receptor and vascular endothelial growth factor with a focus on head and neck tumors.. Curr Opin Oncol.

[6] Hurwitz HI, Fehrenbacher L, Hainsworth JD, Heim W, Berlin J (2005). Bevacizumab in combination with fluorouracil and leucovorin: An active regimen for first-line metastatic colorectal cancer.. J Clin Oncol.

[7] Hack NJ, Wride MC, Charters KM, Kater SB, Parks TN (2000). Developmental changes in the subcellular localization of calretinin.. J Neurosci.

[8] Schwaller B, Durussel I, Jermann D, Herrmann B, Cox JA (1997). Comparison of the Ca2+-binding properties of human recombinant calretinin-22k and calretinin.. J Biol Chem.

[9] Bertschy S, Genton CY, Gotzos V (1998). Selective immunocytochemical localisation of calretinin in the human ovary.. Histochem Cell Biol.

[10] Gotzos V, Wintergerst ES, Musy JP, Spichtin HP, Genton CY (1999). Selective distribution of calretinin in adenocarcinomas of the human colon and adjacent tissues.. Am J Surg Pathol.

[11] Gotzos V, Schwaller B, Gander JC, Bustos-Castillo M, Celio MR (1996). Heterogeneity of expression of the calcium-binding protein calretinin in human colonic cancer cell lines.. Anticancer Res.

[12] Doglioni C, Tos AP, Laurino L, Iuzzolino P, Chiarelli C (1996). Calretinin: A novel immunocytochemical marker for mesothelioma.. Am J Surg Pathol.

[13] Chu AY, Litzky LA, Pasha TL, Acs G, Zhang PJ (2005). Utility of D2-40, a novel mesothelial marker, in the diagnosis of malignant mesothelioma.. Mod Pathol.

[14] Schurmans S, Schiffmann SN, Gurden H, Lemaire M, Lipp HP (1997). Impaired long-term potentiation induction in dentate gyrus of calretinin-deficient mice.. Proc Natl Acad Sci U S A.

[15] Boehning D, Patterson RL, Sedaghat L, Glebova NO, Kurosaki T (2003). Cytochrome c binds to inositol (1,4,5) trisphosphate receptors, amplifying calcium-dependent apoptosis.. Nat Cell Biol.

[16] Mattson MP, Chan SL (2003). Calcium orchestrates apoptosis.. Nat Cell Biol.

[17] Wu Y, Fabritius M, Ip C (2009). Chemotherapeutic Sensitization by Endoplasmic Reticulum Stress: Increasing the Efficacy of Taxane Against Prostate Cancer.. Cancer Biol Ther.

[18] Boyer J, Allen WL, McLean EG, Wilson PM, McCulla A (2006). Pharmacogenomic identification of novel determinants of response to chemotherapy in colon cancer.. Cancer Res.

[19] Longley DB, Wilson TR, McEwan M, Allen WL, McDermott U (2006). c-FLIP inhibits chemotherapy-induced colorectal cancer cell death.. Oncogene.

[20] Boyer J, McLean EG, Aroori S, Wilson P, McCulla A (2004). Characterization of p53 wild-type and null isogenic colorectal cancer cell lines resistant to 5-fluorouracil, oxaliplatin, and irinotecan.. Clin Cancer Res.

[21] Staub E, Groene J, Heinze M, Mennerich D, Roepcke S (2009). An expression module of WIPF1-coexpressed genes identifies patients with favorable prognosis in three tumor types.. J Mol Med.

[22] Chanrion M, Negre V, Fontaine H, Salvetat N, Bibeau F (2008). A gene expression signature that can predict the recurrence of tamoxifen-treated primary breast cancer.. Clin Cancer Res.

[23] Longley DB, Boyer J, Allen WL, Latif T, Ferguson PR (2002). The role of thymidylate synthase induction in modulating p53-regulated gene expression in response to 5-fluorouracil and antifolates.. Cancer Res.

[24] Bunz F, Hwang PM, Torrance C, Waldman T, Zhang Y (1999). Disruption of p53 in human cancer cells alters the responses to therapeutic agents.. J Clin Invest.

[25] Zhang L, Yu J, Park BH, Kinzler KW, Vogelstein B (2000). Role of BAX in the apoptotic response to anticancer agents.. Science.

[26] Wilson TR, McEwan M, McLaughlin K, Le Clorennec C, Allen WL (2009). Combined inhibition of FLIP and XIAP induces Bax-independent apoptosis in type II colorectal cancer cells.. Oncogene.

[27] Liston P, Fong WG, Korneluk RG (2003). The inhibitors of apoptosis: there is more to life than Bcl2.. Oncogene.

[28] D'Orlando C, Fellay B, Schwaller B, Salicio V, Bloc A (2001). Calretinin and calbindin D-28k delay the onset of cell death after excitotoxic stimulation in transfected P19 cells.. Brain Res.

[29] D'Orlando C, Celio MR, Schwaller B (2002). Calretinin and calbindin D-28k, but not parvalbumin protect against glutamate-induced delayed excitotoxicity in transfected N18-RE 105 neuroblastoma-retina hybrid cells.. Brain Res.

[30] Kuźnicki J, Isaacs KR, Jacobowitz DM (1996). The expression of calretinin in transfected PC12 cells provides no protection against Ca(2+)-overload or trophic factor deprivation.. Biochim Biophys Acta.

[31] Kroemer G, Reed JC (2000). Mitochondrial control of cell death.. Nat Med.

