# Supplementary figures and images for: Calbindin 2 (CALB2) Regulates 5-Fluorouracil Sensitivity in Colorectal Cancer by Modulating the Intrinsic Apoptotic Pathway
Source: PLoS One. 2011 May 24;6(5):e20276. doi: 10.1371/journal.pone.0020276 (PMC3101240; doi:10.1371/journal.pone.0020276)

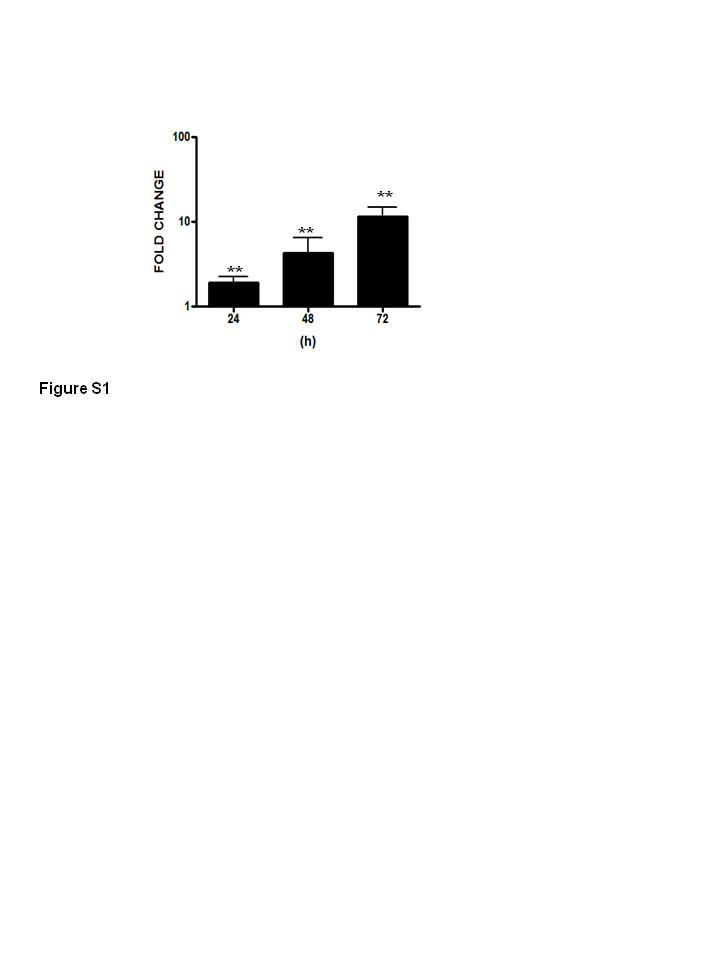

Supplement: Figure S1 — CALB2 gene expression in untreated HCT116 cells. Real-time RT-PCR quantification of CALB2 mRNA levels in untreated p53+/+ HCT116 cells following 24 h, 48 h and 72 h culture. Fold change in expression values are relative to the untreated 0 h control. Error bars represent mean ± SEM, **p<0.01. (TIF) [file pone.0020276.s001.tif]

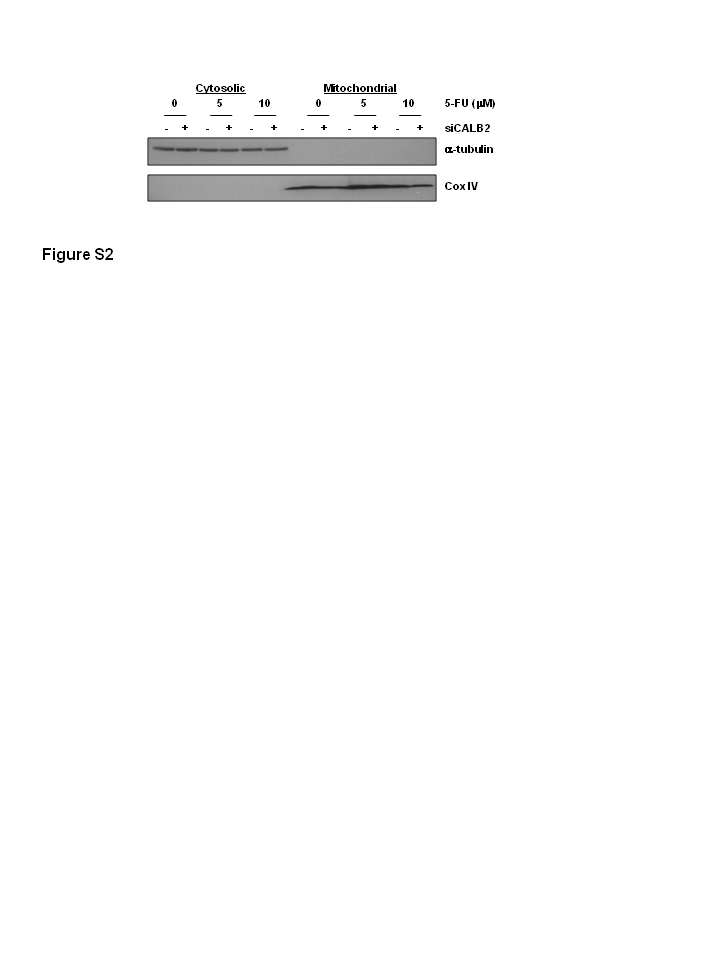

Supplement: Figure S2 — Western blot analysis of CoxIV and α-tubulin levels in p53+/+ HCT116 mitochondrial and cytosolic fractions. (TIF) [file pone.0020276.s002.tif]

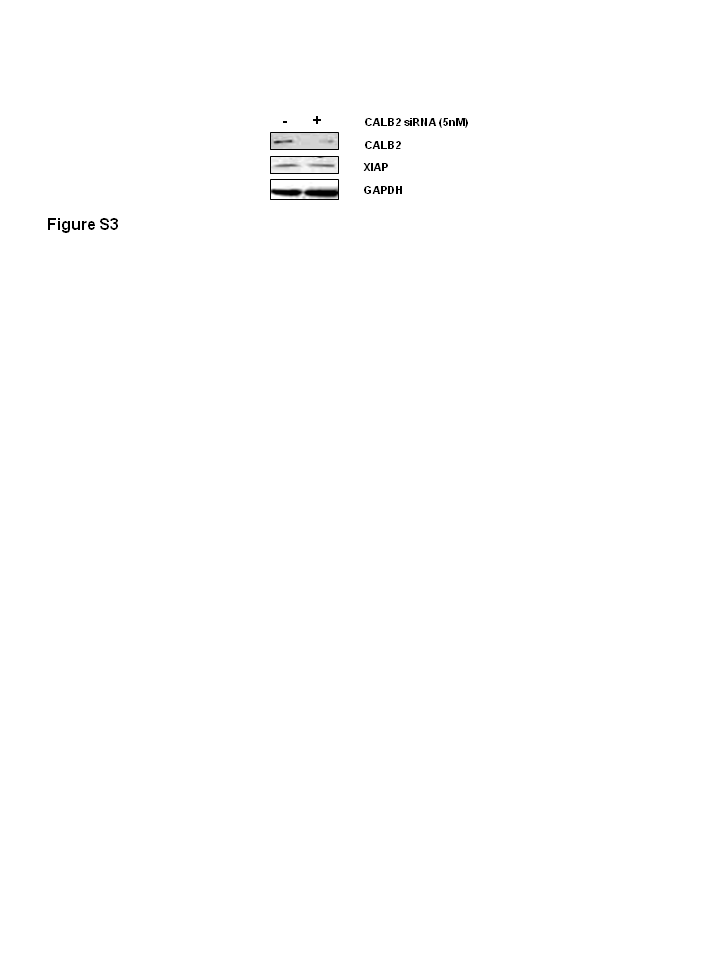

Supplement: Figure S3 — Western blot analysis of XIAP expression following 72 h transfection with 5 nM control siRNA (−) or CALB2 targeted siRNA (+). GAPDH was used as a loading control. (TIF) [file pone.0020276.s003.tif]

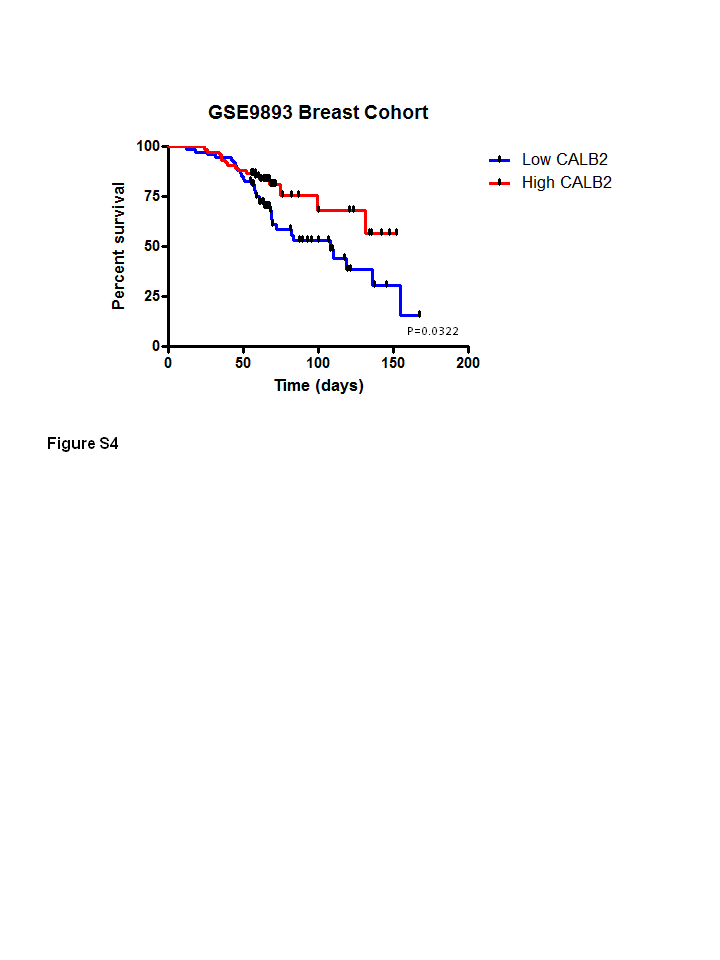

Supplement: Figure S4 — CALB2 expression in breast cancer. Kaplan-Meier survival curve across a breast cancer patient cohort (Dataset: GSE9893, probe ID: 20864; n = 132; HR = 1.861; CI of ratio = 1.054–3.285; p = 0.0322; n = 132). The red line shows patients with high CALB2 expression and the blue line shows patients with low CALB2 expression. High and low level expression cut-points were based on median CALB2 expression. (TIF) [file pone.0020276.s004.tif]
