# Supplementary material for: Calbindin 2 (CALB2) Regulates 5-Fluorouracil Sensitivity in Colorectal Cancer by Modulating the Intrinsic Apoptotic Pathway
Source: PLoS One. 2011 May 24;6(5):e20276. doi: 10.1371/journal.pone.0020276 (PMC3101240; doi:10.1371/journal.pone.0020276)
Supplement: Table S1 — Identification of de-regulated pathways associated with 5-FU resistance by pathway analysis. KEGG pathway analysis was used to identify pathways associated with 5-FU resistance from the 5-FU in vitro genelists: (1) genes induced by 5-FU in the sensitive HCT116 parentals (inducible parental), (2) genes induced by 5-FU in the 5-FU-resistant HCT116 sub-line (inducible resistant) and (3) constitutively de-regulated genes in the 5-FU-resistant sub-line compared to the sensitive parentals (constitutively deregulated). All pathways contained ≥5 genes passing a 1.5 fold change and t-test (p<0.05). (DOCX) [file pone.0020276.s005.docx]

**Table S1 Identification of de-regulated pathways associated with 5-FU resistance by pathway analysis.** KEGG pathway analysis was used to identify pathways associated with 5-FU resistance from the 5-FU *in vitro* genelists: (1) genes induced by 5-FU in the sensitive HCT116 parentals (inducible parental), (2) genes induced by 5-FU in the 5-FU-resistant HCT116 sub-line (inducible resistant) and (3) constitutively de-regulated genes in the 5-FU-resistant sub-line compared to the sensitive parentals (constitutively deregulated). All pathways contained ≥5 genes passing a 1.5 fold change and t-test (p<0.05).

| **Parental inducible genelist pathways** | **No of genes** | **Constitutively deregulated genelist** **pathways** | **No of genes** | **Inducible resistant** genelist **pathways** | **No of genes** |
| --- | --- | --- | --- | --- | --- |
| Adipocytokine signaling pathway | 5 | ABC transporters - General | 5 | Adherens junction | 13 |
| Alanine and aspartate metabolism | 6 | Adherens junction | 6 | Alanine and aspartate metabolism | 8 |
| Aminophosphonate metabolism | 6 | Adipocytokine signaling pathway | 7 | Aminoacyl-tRNA biosynthesis | 8 |
| Amyotrophic lateral sclerosis (ALS) | 5 | Antigen processing and presentation | 9 | Apoptosis | 7 |
| Androgen and estrogen metabolism | 6 | Apoptosis | 5 | Axon guidance | 11 |
| Apoptosis | 7 | Arginine and proline metabolism | 5 | Calcium signaling pathway | 7 |
| Biosynthesis of steroids | 6 | Axon guidance | 14 | Cell Communication | 6 |
| Butanoate metabolism | 7 | Butanoate metabolism | 5 | Cell cycle | 13 |
| Calcium signaling pathway | 8 | Calcium signaling pathway | 9 | Focal adhesion | 13 |
| Cell cycle | 21 | Cell adhesion molecules (CAMs) | 7 | Gap junction | 7 |
| Chronic myeloid leukemia | 7 | Cell Communication | 5 | Glycine, serine and threonine metabolism | 9 |
| Citrate cycle (TCA cycle) | 9 | Cell cycle | 11 | Insulin signaling pathway | 12 |
| Colorectal cancer | 11 | Chronic myeloid leukemia | 5 | Leukocyte transendothelial migration | 11 |
| Cytokine-cytokine receptor interaction | 5 | Colorectal cancer | 5 | MAPK signaling pathway | 14 |
| DNA polymerase | 6 | Complement and coagulation cascades | 5 | Pathogenic Escherichia coli infection - EHEC | 11 |
| Epithelial cell signaling in Helicobacter pylori infection | 6 | Cytokine-cytokine receptor interaction | 9 | Pathogenic Escherichia coli infection - EPEC | 11 |
| Fatty acid metabolism | 7 | Focal adhesion | 10 | Proteasome | 9 |
| Fc epsilon RI signaling pathway | 6 | Glutamate metabolism | 6 | Purine metabolism | 12 |
| Focal adhesion | 16 | Glycerolipid metabolism | 6 | Pyrimidine metabolism | 12 |
| Fructose and mannose metabolism | 14 | Glycerophospholipid metabolism | 6 | Regulation of actin cytoskeleton | 16 |
| Galactose metabolism | 6 | Hematopoietic cell lineage | 6 | Ribosome | 5 |
| Gap junction | 5 | Insulin signaling pathway | 8 | T cell receptor signaling pathway | 5 |
| Glioma | 7 | Jak-STAT signaling pathway | 14 | Tight junction | 14 |
| Glycan structures - biosynthesis 1 | 6 | Lysine degradation | 7 | Wnt signaling pathway | 8 |
| Glycan structures - biosynthesis 2 | 6 | MAPK signaling pathway | 12 |  |  |
| Glycerophospholipid metabolism | 8 | Melanogenesis | 5 |  |  |
| Glycine, serine and threonine metabolism | 7 | Melanoma | 6 |  |  |
| Glycolysis Gluconeogenesis | 12 | Methionine metabolism | 5 |  |  |
| Glycosylphosphatidylinositol(GPI)-anchor biosynthesis | 5 | Neuroactive ligand-receptor interaction | 5 |  |  |
| GnRH signaling pathway | 5 | Nicotinate and nicotinamide metabolism | 6 |  |  |
| Histidine metabolism | 10 | Notch signaling pathway | 5 |  |  |
| Insulin signaling pathway | 20 | Olfactory transduction | 5 |  |  |
| Lysine degradation | 8 | One carbon pool by folate | 7 |  |  |
| MAPK signaling pathway | 19 | Oxidative phosphorylation | 11 |  |  |
| Melanoma | 7 | Pancreatic cancer | 11 |  |  |
| Methane metabolism | 6 | PPAR signaling pathway | 9 |  |  |
| Neurodegenerative Disorders | 8 | Purine metabolism | 17 |  |  |
| N-Glycan biosynthesis | 7 | Pyrimidine metabolism | 10 |  |  |
| Nicotinate and nicotinamide metabolism | 7 | Regulation of actin cytoskeleton | 8 |  |  |
| Nitrobenzene degradation | 5 | Renal cell carcinoma | 8 |  |  |
| Oxidative phosphorylation | 17 | Starch and sucrose metabolism | 5 |  |  |
| Pancreatic cancer | 10 | TGF-beta signaling pathway | 8 |  |  |
| Pentose phosphate pathway | 10 | Thyroid cancer | 5 |  |  |
| Propanoate metabolism | 5 | Tight junction | 7 |  |  |
| Proteasome | 13 | Toll-like receptor signaling pathway | 7 |  |  |
| Purine metabolism | 23 | Tryptophan metabolism | 11 |  |  |
| Pyrimidine metabolism | 19 | Ubiquinone biosynthesis | 5 |  |  |
| Pyruvate metabolism | 9 | Ubiquitin mediated proteolysis | 8 |  |  |
| Regulation of actin cytoskeleton | 15 | Wnt signaling pathway | 8 |  |  |
| Renal cell carcinoma | 7 |  |  |  |  |
| Selenoamino acid metabolism | 8 |  |  |  |  |
| Starch and sucrose metabolism | 11 |  |  |  |  |
| Tight junction | 6 |  |  |  |  |
| Tryptophan metabolism | 16 |  |  |  |  |
| Tyrosine metabolism | 8 |  |  |  |  |
| Ubiquitin mediated proteolysis | 9 |  |  |  |  |
| Urea cycle and metabolism of amino groups | 5 |  |  |  |  |
| Valine, leucine and isoleucine degradation | 5 |  |  |  |  |
| VEGF signaling pathway | 6 |  |  |  |  |
| Wnt signaling pathway | 12 |  |  |  |  |
